# Plant Genes Benefitting Aphids—Potential for Exploitation in Resistance Breeding

**DOI:** 10.3389/fpls.2019.01452

**Published:** 2019-11-13

**Authors:** Inger Åhman, Sung-Yong Kim, Li-Hua Zhu

**Affiliations:** Department of Plant Breeding, Swedish University of Agricultural Sciences, Alnarp, Sweden

**Keywords:** aphid, susceptibility gene, omics, plant resistance, plant defense, gene editing, CRISPR/Cas9, RNAi

## Abstract

Aphids are phloem sap-feeding insects common as pests in various crops. Here we review 62 omics studies of aphid/plant interactions to search for indications of how aphids may manipulate the plants to make them more suitable as hosts, i.e. more susceptible. Our aim is to try to reveal host plant susceptibility (*S*) genes, knowledge which can be exploited for making a plant more resistant to its pest by using new plant breeding techniques to knock out or down such *S* genes. *S* genes may be of two types, those that are involved in reducing functional plant defense and those involved in further increasing plant factors that are positive to the aphid, such as facilitated access to food or improved nutritional quality. Approximately 40% of the omics studies we have reviewed indicate how aphids may modify their host to their advantage. To exploit knowledge obtained so far, we suggest knocking out/down candidate aphid *S* genes using CRISPR/Cas9 or RNAi techniques in crops to evaluate if this will be sufficient to keep the aphid pest at economically viable levels without severe pleiotropic effects. As a complement, we also propose functional studies of recessively inherited resistance previously discovered in some aphid–crop combinations, to potentially identify new types of *S* genes that later could be knocked out or down also in other crops to improve their resistance to aphids.

## Introduction

Aphids are phloem sap-feeding insects and most cultivated plant species are hosts of one or more aphid species. Aphids damage plants mainly by plant nutrient withdrawal, by virus transmission, and *via* indirect reduction of photosynthesis due to sooty mould growing in their excreta. Some species also change form and color of the plant tissues locally. Aphids can have complicated life cycles alternating between primary and secondary hosts, reproduce sexually or asexually, and be winged (alate) or without wings (apterous). Crops are invaded by alate aphids which give rise to successive apterous generations parthenogenetically (Blackman and Eastop, 1984). Aphids in these clonal colonies are sedentary if weather conditions are favorable, if host plant quality is high and if there is minimal disturbance by natural enemies. Insects with this life style have evolved so that they can modify their host plants locally to their own benefit (Walling, 2008; Giordanengo et al., 2010; Zust and Agrawal, 2016). Here we review such evidence of host manipulation and suggest how this knowledge can be used to breed plants which are less suitable as aphid hosts.

The number of aphid species in the family Aphididae is approximately 4 700 and circa 10% of those occur on crops (Blackman and Eastop, 2007). Most aphid species are specialized in their host range. It is common for aphids to only use plant species within a plant family as hosts, or species from two distinct families if aphids are host alternating (Blackman and Eastop 1984). This high degree of specialization requires that aphids have the ability to distinguish hosts from non-hosts using their sensory organs (Pettersson et al., 2007). Despite this host specialisation aphids have certain feeding characteristics in common. After alighting on a plant, aphids start probing with their stylet-shaped mouthparts, on the plant surface and in the plant tissue. The stylet is predominantly pushed in between plant cells, but during its way to the phloem most plant cells along the route are briefly punctured (Pettersson et al., 2007). This is believed partly to take place for the aphids to localize the stylet in relation to phloem tissue and to assess host status, but also to inject watery saliva to improve host quality. Watery saliva is also injected into the phloem, before the phloem sap is taken up. During ingestion, watery saliva is produced as well, transported with the phloem sap into the food canal (Pettersson et al., 2007). Over the whole plant probing period, gelling saliva is also produced. This type of saliva forms a protective sheet around the stylet and seals punctured plant cells. The chemical content of gelling saliva is more conserved between aphid species than is the content of the watery saliva. The main compound in gelling saliva is a sheath-forming protein, and probably there are also cell wall-degrading proteins and oxidases (van Bel and Will, 2016). The watery saliva contains various compounds like carbohydrates, phospholipids and proteins, of which many are in the categories of Ca^2+^-binding, proteolytic and oxidising proteins and effectors (Thompson and Goggin, 2006; Giordanengo et al., 2010). Effectors are compounds that interact with the host plant and thereby change plant function and structures in various ways (Rodriguez and Bos, 2013). Several proteins from aphid watery saliva have been shown to promote or reduce aphid performance in a species-specific way. In compatible plant interactions, aphid performance may be promoted by effector-driven downregulation of functional plant defense or by regulation of plant factors that are positive to the aphid (Giordanengo et al., 2010; van Bel and Will, 2016; Mondal, 2017).

The knowledge about aphid-induced plant responses has increased dramatically during recent years. There are now many pairs of aphid species/plant species combinations where “global” plant gene regulations have been studied, mainly at the transcriptional level, but to some extent also at the protein and metabolite levels. Aphid-induced plant genes may change the way the plant functions as an aphid host, to the better or to the worse. Approximately half of the 62 studies presented in **Table 1** have included both susceptible and resistant plant genotypes, with the aim to distinguish differences between these two categories of plants in order to better understand mechanisms of plant resistance. This might in turn be used for more precise selection of resistant plants in plant breeding programs. In the present review we instead aim at emphasizing similarities in gene regulation between susceptible plants subjected to aphid feeding, keeping in mind that there might also be aphid/plant-specific interactions. This knowledge may be applied in breeding programs with the aim to reduce susceptibility rather than to increase resistance to aphids.

**Table 1:** Global studies of plant genes as DNA contigs, RNA sequences, probes, proteins, or metabolites regulated by aphid feeding.

Plant species	Aphid species	Reference	Method	No. of “genes”	Plant type	Comments
Alfalfa	*Therioaphis trifolii*	Tu et al., 2018	RNAseq	184,892	1 Res/1 Sus	SA, JA, and flavonoid pathways induced 72 h
	*Acyrthosiphon pisum*	Sanchez-Arcos et al., 2019	Metabolomics	8,455	2 Res/2 Sus species	SUS: Compounds in alfalfa downregulated by alfalfa biotype but not by clover and pea biotype of the aphid, e.g. a triterpene saponin 48 h
Apple	*Dysaphis plantaginea*	Qubbaj et al., 2005	cDNA-AFLP		1 Res/1 Sus	Signaling and photosynthesis genes more regulated in Res 72 h
*Arabidopsis*	*Myzus persicae* *Brevicoryne brassicae*	Moran et al., 2002	Micro- and macroarray	105	1 Sus	Oxidative stress, PR proteins, signaling SUS: nutrient sink-related sugar symporter (SUS): *β*-1,3-glucanase gene upregulated 72, 96 h
	*Myzus persicae*	De Vos et al., 2005	Microarray	23,750	1 Sus	SUS: metabolism genes downregulated 72 h
	*Myzus persicae*	Couldridge et al., 2007	Microarray	22,500	1 Sus	SUS: arabinogalactan protein gene downregulated, photosynthesis-related gene upregulated indicative of sink 2, 36 h
	*Myzus persicae* *Brevicoryne brassicae*	Kusnierczyk et al., 2007	Microarray	2,158	3 Sus 1 Sus/1 moderately Sus/1Res	SUS: downregulation of genes relating to hydrolysis of glucosinolates 72 h
	*Brevicoryne brassicae*	Kusnierczyk et al., 2008	Microarray	26,604	1 moderately Sus + 1 mutant	Camalexin related to resistance 6, 12, 24, 48 h
	*Myzus persicae* saliva	De Vos & Jander 2009	Microarray	22,500	1 Sus	High overlap between aphid- and saliva-induced transcripts. Defense induced in infested leaves, not in systemic 24 h
	*Brevicoryne brassicae*	Kusnierczyk et al., 2011	Microarray	26,604	1 Sus + 2 mutants	JA signaling related to defense 72 h
	*Myzus persicae*	Bricchi et al., 2012	Microarray	38,463	1 Sus	SUS: Downregulated JA pathway, secondary compound transporters, flavonoid synthesis, and chitin-responsive transcription factors; increased cell wall loosening 5 h
	*Brevicoryne brassicae*	Kutyniok and Muller, 2012	Metabolomics		1 Sus	Low aphid density caused no changes in JA and SA levels, aliphatic glucosinolates were reduced 3 days
	*Brevicoryne brassicae*	Barah et al., 2013	Microarray	26,604	1 Sus	Ethylene-related genes upregulated, aliphatic glucosinolate genes downregulated 72 h
	*Myzus persicae*	Kerchev et al., 2013	Microarray	43,803	1 Sus + mutants	Infested and non-infested leaves compared SUS: ABA and redox-responsive transcription factor 6, 24, 48 h
	*Myzus persicae* *Brevicoryne brassicae*	Appel et al., 2014	Microarray	26,090	1 Sus	Little overlap between gene regulation by the two aphid species 1 week infestation then analyzed 6, 24 h after aphid removal
	*Myzus persicae Myzus cerasi Rhopalosiphum padi*	Jaouannet et al., 2015	Microarray	43,408	1 Host poor-host non-host + mutants	Genes that show opposite regulation in host, non-host, or poor-host: SUS *M.p.*: six genes with various functions SUS *R.p.*: ABA-responsive late embryogenesis gene 0, 3, 6, 24 h
	*Myzus persicae*	Truong et al., 2015	Proteomics	574	1 Sus	Several photosynthesis proteins regulated 3 days
Barley	*Diuraphis noxia*	Gutsche et al., 2009	Microarray	> 21,000	1 Tol/1 Sus	No differences in aphid performance. ROS scavenging more efficient in Tol 3 h, 3 days, 6 days
	*Rhopalosiphum padi*	Delp et al., 2009	Microarray	> 21,000	2 moderately Res/2 Sus	SUS: two sugar transporters upregulated in all four and one in the two Sus indicating aphids inducing leaf sink (SUS): certain *β*-1,3-glucanases upregulated in all four or specific to the two Sus 48 h
	*Sitobion avenae*	Zytynska et al., 2016	Microarray	22,740	1 Sus	> 1,000 transcripts differentially regulated comparing one aphid biotype to another, altogether four biotypes 5 days
	*Rhopalosiphum padi* *Myzus persicae* *Myzus cerasi*	Escudero-Martinez et al., 2017	Microarray	60,000	1 Host Poor-host Non-host	Poor-host interaction led to stronger induction than host interaction 3, 24 h
Barrel medic	*Acyrthosiphon pisum*	Sun et al., 2018	Proteomics	1,592	1 Res/1 Sus NILs	*R*-gene defense relates to heat shock protein and its chaperon, and JA (SUS): JA-related proteins are suppressed in Sus plant, *β*-1,3-glucanase more prevalent in Sus than in Res 24 h
Black mustard	*Brevicoryne brassicae*	Broekgaarden et al., 2011	Microarray	26,173	Sus population	(SUS): Suppression of *O*-methyl transferase might lead to less lignin and thereby looser cell wall. Repressor of the ET defense pathway up-regulated as well as an ET suppressor gene 48 h
	*Brevicoryne brassicae*	Ponzio et al., 2017	Metabolomics		Sus population	Local effects stronger than systemic effects. Locally aphids induced various sugars 72 h
Cabbage	*Brevicoryne brassicae*	Broekgaarden et al., 2008	Microarray	26,173	1 moderately Res/1 Sus	SUS: Upregulation of aquaporin genes might increase access to nutrients in phloem 48 h
Celery	*Myzus persicae*	Divol et al., 2005	Macroarray SSH	891	1 Sus	Systemic response in phloem tissue analyzed SUS: Thiamine (vitamin B_1_) biosynthesis gene strongly upregulated, cell wall and water channel genes regulated for control of turgor pressure 3, 7 days
Corn	*Rhopalosiphum maidis*	Tzin et al., 2015	RNASeq Metabolomics	20,000	1 Sus + mutants	SUS: Temporal gene regulation patterns ruled by the aphid, ABA-related genes upregulated to maintain water potential, oxylipin genes up-regulated to provide lipid nutrients, cytokinin-related genes downregulated to promote senescence to release amino acids 2, 4, 8, 24, 48, 96 h
Cotton	*Aphis gossypii*	Dubey et al., 2013	RNASeq	14,810	1 Sus	SUS: genes regulated to increase sugar and amino acid concentration in phloem, to advance senescence, to hinder sieve tube clogging and to suppress ET defense pathway 2, 24 h
	*Aphis gossypii*	Eisenring et al., 2018	Metabolomics		1 Sus	Systemically induced leaves investigated. SA level reduced 7–8 days
Cucumber	*Aphis gossypii*	Liang et al., 2015	RNASeq		1 Res	Several candidate genes for aphid resistance 0, 2, 4, 6, 8 days
Pea	*Acyrthosiphon pisum*	Carrillo et al., 2014	Proteomics		1 moderately Res/1 Sus	SUS: Amino acid synthesis, photosynthesis and carbohydrate metabolism proteins abundant in infested Sus tissue 24, 84 h
Potato	*Myzus persicae,* *Macrosiphum euphorbiae*	Alvarez et al., 2013	Microarray	3,570	1 Res wild species *Solanum stoloniferum* 1 Sus	SUS: genes regulated to switch the tissue from source to sink; transporters, protein metabolism 96 h
	*Myzus persicae*	Alvarez et al., 2014	Microarray	3,570	1 “Res” = young leaves/1 Sus = old leaves	SUS: SA pathway genes upregulated to hinder JA induction to which aphid is less tolerant, genes regulated to switch the tissue from source to sink, genes for sugar and amino acid transport and mobilization upregulated, downregulation of secondary compound-related genes 96 h
Rose	No species name	Muneer et al., 2018	Proteomics		2 Res (Tol)?/2 Sus	Aphid-induced tissue compared between Res and Sus (only 9 proteins analyzed) Induction time unknown
Sorghum	*Schizaphis graminum*	Zhu-Salzman et al., 2004	Microarray SSH	672	1 Sus	SUS: Induction of SA signaling suppresses JA pathway which is effective as aphid defense (SUS): *β*-1,3-glucanase and nitrate reductase genes upregulated 48 h
	*Schizaphis graminum*	Park et al., 2006	Microarray SSH		1 Res/1 Sus	(SUS): *β*-1,3-glucanase genes more upregulated in Sus 72 h
	“Sorghum aphids”	Chang et al., 2012	AFLP		1 Res/1 Sus + F2 population	Defense-related genes identified among the 65 differentially expressed 0, 24, 48, 72 h (+2 h)
	*Melanaphis sacchari*	Kiani and Szczepaniec, 2018	RNAseq		1 Res/1 Sus	SUS: JAZ genes upregulated which are negative regulators of JA-related genes 24 h
	*Melanaphis sacchari*	Tetreault et al., 2019	RNASeq		1 Res/1 Sus	JA- and ET-related genes upregulated and photosynthesis and peroxidase genes downregulated in Sus 5, 10, 15 days (late sampling)
Soybean	*Aphis glycines*	Li et al., 2008	Microarray	18,000	1 Res/1 Sus	95 genes specifically regulated in Sus 6, 12 h
	*Aphis glycines*	Studham and MacIntosh, 2013	Microarray	22,763	1 Res/1 Sus	SUS: JA, ET, and later especially ABA defense pathway genes upregulated to hinder SA synthesis. Gene-silencing regulator upregulated in Sus 24 h, 7 days
	*Aulacorthum solani*	Sato et al., 2013	Metabolomics		1 Res/1 Sus	Phenylpropanoid synthesis precursors more abundant in Sus at 6 h 0, 6, 12, 24, 48, 96 h
	*Aphis glycines*	Prochaska et al., 2015	RNASeq		1 Tol/1 Sus	(SUS): Nitrate reductase upregulated in Sus and downregulated in Tol 5, 15 days
	*Aphis glycines*	Brechenmacher et al., 2015	RNASeq Proteomics	3,445 proteins	1 Res/1 Sus	NILS with and without *Rag2* gene in search of resistance gene candidates 0, (4), 8, 24, 48 h
	*Aphis glycines*	Lee et al., 2017	RNASeq		1 Res/1 Sus	NILS with and without *Rag5* gene in search of resistance gene candidates 0, 6, 12, 48 h
	*Aphis glycines*	Hohenstein et al., 2019	Microarray	22 763	1 Res/1 Sus	ABA signaling absent interpreted as ABA Sus induction by the aphid overcome by the plant by day 21 after infection SUS: Alternatively, the aphid has stopped ABA induction to allow the plant to produce deterrents to advance aphid migration 21 days
Tobacco	*Myzus nicotianae*	Voelckel et al., 2004	Microarray	240	1 Sus wild species *Nicotiana attenuata*	Local and systemic leaves with and without aphids compared SUS: Glutamate synthase higher expression in local (sink) leaves, downregulation of peroxide-generating germin gene locally 2 days
	*Myzus nicotianae*	Heidel and Baldwin, 2004	Microarray	481	1 Sus wild species *Nicotiana attenuata*	Only systemic effect analyzed. (SUS): Nitrogen-related genes upregulated 72 h
Tomato	*Macrosiphum euphorbiae*	Rodriguez-Saona et al., 2010	Microarray	13 440	1 Sus	Only systemic effects analyzed SUS: Many JA pathway genes suppressed, N/C ratio increased 5 days
	*Macrosiphum euphorbiae*	Coppola et al., 2013	Microarray Proteomics	21,000	1 Sus	SUS: SA and ET pathway genes upregulated may hinder JA upregulation, genes for N transport upregulated 24, 48, 96 h
	*Myzus persicae*	Errard et al., 2015	Metabolomics		1 Sus	Volatile and non-volatile substances; methyl salicylate induced, among many others 3 weeks
Wheat	*Diuraphis noxia*	Botha et al., 2006	Microarray SSH	256	1 Res	Gene regulation in genotype with *Dn1* resistance 0, 2, 5, 8 days
	*Diuraphis noxia*	Boyko et al., 2006	SSH		Res/Sus 10/10 F_2:3_ bulked	Gene regulation interpreted in relation to antibiosis and plant tolerance 48 h
	*Diuraphis noxia*	Botha et al., 2010	Microarray	55,052	1 Res/1 Sus	Gene regulation by two aphid biotypes on a *Dn7-*containing cultivar and a susceptible cultivar 5, 48 h
	*Diuraphis noxia*	Smith et al., 2010	Microarray	55,052	Res/Sus 10/10 F_2:4_bulked	Many genes upregulated earlier in Res than in Sus. Auxin-related genes upregulated only in Sus 24 h
	*Diuraphis noxia*	Liu et al., 2011	EST RT-qPCR	10 defense 16 meta-bolic	1 Res = Sus	One aphid biotype compatible with plant genotype and one incompatible. Local and systemic effects analyzed. More gene regulation in local tissue. No indications of source/sink effects. JA-related defense 1, 3, 6, 24 h
	*Sitobion avenae*	Ferry et al., 2011	Proteomics	Ca. 500	1 Sus	Local and systemic effects analyzed (SUS): More metabolism proteins regulated in systemic than in local tissue, JA-regulated agglutinin downregulated in local tissue at both time points, more SA- than JA-regulated proteins indicates suppression of JA pathway 24 h, 8 days
	*Schizaphis graminum*	Reddy et al., 2013	Microarray	55,052	Res/Sus 8/8 _F8_ RILs bulked	Bulk with and without *Gb3*-gene SUS: Facilitated stylet penetration and sustained feeding by aphid regulation of cell wall and callose decomposition genes such as cellulose synthases, pectinases, and glucanases 0, 24, 48 h
	*Diuraphis noxia*	Botha et al., 2014	cDNA AFLP Microarray		1 Sus/3 NILs	Tugela and Tugela with *Dn1* (antibiosis *via* HR), *Dn2* (tolerance *via* photosynthesis repair), *Dn5* (antixenosis *via* volatiles). 2, 6, 12, 24, 48 h
	*Sitobion avenae*	Guan et al., 2015	Proteomics	Ca. 200	1 Res/1 Sus *Triticum monococcum*	Local and systemic effects analyzed. (SUS): Downregulated proteins not shown since considered not relevant for Res as they may result from aphid manipulation 24 h, 8 days
	*Rhopalosiphum padi*	Greenslade et al., 2016	Metabolomics		3 partially Res (*Triticum monococcum*)/1 Sus wheat	No systemic effect when non-infested part of the leaves is compared with the infested 24 h
	*Rhopalosiphum padi* *Schizaphis graminum* *Sitobion avenae*	Shavit et al., 2018	Metabolomics	376	1 Sus partially Res Res durum wheat	Metabolite changes induced by *S. graminum* differed from those of the other two aphid species interpreted as related to the leaf symptoms induced by *S. graminum* 96 h

*Part of the studies infers aphid-induced susceptibility. If authors suggest a susceptibility function it is here remarked as “SUS:” in the “Comments” column, and “(SUS):” if it is interpreted as such by us. Time points for sampling after infestation are also noted.*

RNASeq, RNA sequencing; cDNA, complementary DNA; AFLP, Amplified fragment length polymorphism; SSH, Subtractive suppression hybridisation; EST, Expressed sequence tag; RT, Reversed transcript; qPCR, quantitative polymerase chain reaction; Res, Resistant genotype; Sus, Susceptible genotype; Tol, Tolerant genotype; NILs, Near isogenic lines; RILs, Recombinant inbred lines; SA, Salicylic acid; JA, Jasmonic acid; ET, Ethylene and ABA, Abscisic acid hormonal pathways; ROS, Reactive oxygen species; PR, Pathogen-related; HR, Hypersensitive response; M.p., Myzus persicae; R.p., Rhopalosiphum padi.

In recent years two new plant breeding approaches offer possibilities to exploit such knowledge. RNA interference (RNAi) may knock down expression of susceptibility (*S*) genes and site-directed mutagenesis techniques such as CRISPR/Cas9 may knock out such genes. Since these new breeding techniques are best suited to reduce gene expression levels, we have focussed on candidate *S* genes that are upregulated by aphids, even though also plant genes that are downregulated by aphids may facilitate aphid host acceptance and performance. Furthermore, there must be numerous plant genes that through their constitutive expression favor the aphid. However, it is very difficult to distinguish which of these are the most important *S* genes and most of them are likely to be essential also for plant fitness. Apart from using omics information to try to discern *S* genes, there is also the possibility to investigate the molecular mechanisms behind previously detected resistance to aphids that is inherited recessively. Potentially such resistance may be due to *S* genes that have been spontaneously mutated in the host.

## Aphid-Induced Plant Genes

Candidate genes for induced susceptibility are little addressed in the studies of global gene expressions in the aphid host plants (**Table 1**). On the contrary, genes are often referred to as defense genes even in studies of aphids on susceptible hosts. One of the difficulties to infer a gene function in induced susceptibility is that susceptibility is often a quantitative trait. An induced reduction in functional plant defense makes the plant somewhat more susceptible and vice versa. Another difficulty is that both up and down gene regulation may lead to increased host susceptibility depending on the function of the gene product. Yet another difficulty is that RNA transcript abundances are rarely linearly related to the quantities of the metabolic products with potential effects on aphid performance. Partly, this discrepancy is due to timing since gene regulation and expression precede metabolism. This dynamic nature of the induction requires sampling and analyzing at many time points, which is costly. Other reasons for discrepancies between transcripts and metabolites are the complicated interactions between gene products and limitations in access to substrates in the metabolic pathways. Despite difficulties like this, in ca. 40% of the global expression studies listed in **Table 1**, the authors infer certain gene classes, proteins or metabolites to have a susceptibility function. Here we summarize the results from these studies. Moreover, we include also those global expression studies where susceptibility is not discussed, for providing information regarding other potential target genes for future studies. In the following, the susceptibility concept is discussed in terms of how it relates to aphid-induced changes in plant defense pathways (see *Plant Hormonal Induction in Relation to Functional Plant Defense*), local and systemic induction patterns (*Local and Systemic Induction Patterns Influencing Aphid Performance*), and improvements of food accessibility (*Aphid Modification of Food Accessibility*) and quality (*Aphid Modification of Food Quality*), mainly with examples from the studies listed in **Table 1**.

### Plant Hormonal Induction in Relation to Functional Plant Defense

Plant receptors can detect invaders such as herbivorous insects and induce defense signaling pathways, mainly involving the salicylate (SA) and the jasmonate (JA) pathways, modified primarily by the ethylene (ET), abscisic acid (ABA), and gibberellic acid (GA) related ones. Whereas chewing insects predominantly induce the JA pathway genes, aphids commonly induce genes related to the SA pathway. Since aphids, in general, have been shown to be more sensitive to plant defense involving the JA signaling pathway, SA induction is considered to be a way for aphids to deceive the plant because cross-talk between hormonal pathways hinders the SA-induced plant to fully induce the JA and ET pathways (Thompson and Goggin 2006; De Vos et al., 2007; Goggin, 2007; Walling, 2008). Indications of this SA–JA antagonism have been found in the global expression studies of *Acyrthosiphon pisum* Harris on susceptible barrel medic (Sun et al., 2018), *Myzus persicae* Sulzer on potato (Alvarez et al., 2014), *Schizaphis graminum* Rondani on susceptible sorghum (Zhu-Salzman et al., 2004), *Melanaphis sacchari* Zehntner on sorghum (Kiani and Szczepaniec, 2018), *Macrosiphum euphorbiae* Thomas on susceptible tomato (Coppola et al., 2013), and *Sitobion avenae* Fabricius on susceptible wheat (Ferry et al., 2011). However, in the study by Bricchi et al. (2012), there was no indication of SA-dependant crosstalk and yet many genes in the JA pathway were downregulated by *M. persicae* on susceptible *Arabidopsis* at the early sampling of 5 h after aphid infestation. A similar result was found with *M. euphorbiae* in susceptible tomato by Rodriguez-Saona et al. (2010). Perhaps contrary to expectations, JA-defense type oxylipins induced by *M. persicae* in *Arabidopsis* produced in roots facilitated leaf infestation by the aphid (Nalam et al., 2012).

There are thus reasons to believe that the explanation for aphid-induced plant susceptibility *via* SA/JA hormonal competition is somewhat simplified. Also ABA-signaling is inferred to be manipulated by aphids to negatively regulate plant defense; in the non-host relationship of *Rhopalosiphum padi* L. on *Arabidopsis* (Jaouannet et al., 2015) as well as in the host relationship of *M. persicae* on *Arabidopsis* (Kerchev et al., 2013; Hillwig et al., 2016) and by *Aphis glycines* Matsumura on susceptible soybean (Studham and MacIntosh, 2013). In addition, the authors of the soybean study speculated that also ET-related gene induction might be a way of blocking the JA induction, since *A. glycines* also upregulated ET-related genes but the aphid’s performance was not affected by artificial ET induction. The finding by Mantelin et al. (2009) that impaired ET signaling or biosynthesis decreases tomato susceptibility to *M. euphorbiae* might support this. However, there are also studies that do not lend support to this aphid-induced plant hormone regulatory role of ET. *Brevicoryne brassicae* L. upregulates a negative regulator gene for ET signaling and an ET suppressor gene whereas SA- and JA-related genes are unaffected in its host black mustard (Broekgaarden et al., 2011). ET-related genes are suppressed also by *Aphis gossypii* Glover in susceptible cotton (Dubey et al., 2013). Furthermore, Zhu et al. (2018) found that *M. persicae* upregulated the transcription factor MYB102 in *Arabidopsis* which in turn upregulated ET biosynthesis. This led to increased host susceptibility but with no evidence for other hormones to be involved. Thus, the relations between plant hormonal regulation by aphids and aphid performance are complicated and perhaps aphid/plant specific.

The SA defense pathway is typically induced by biotrophic pathogens and may lead to local cell death hindering the pathogen to infest and proliferate in the plant. However, such a hypersensitive response is not always seen when aphids upregulate the SA pathway in resistant plants (Thompson and Goggin, 2006). There are so far only three aphid resistance genes cloned and sequenced. *Mi-1* in tomato confers resistance to certain biotypes of *M. euphorbiae. Vat* confers resistance to *A. gossypii* in melon (Dogimont et al., 2010) and *SLI1* to *M. persicae* in *Arabidopsis* (Kloth et al., 2017). *Mi-1* and *Vat* are of the type nucleotide-binding-site leucine-rich repeat (NBS-LLR) known to be involved in recognition of specific effectors secreted by plant-associated organisms and commonly denoted as *R* genes. Upon recognition they trigger plant hormonal defense signaling, which in turn activates plant defense. Interestingly, *Mi-1* mostly relies upon the SA signaling (Dogimont et al., 2010), challenging the ideas that induction of the SA signaling pathway will hinder the plant to embark supposedly efficient JA-dependant plant defense against aphids and that SA defense is not functional against aphids. In line with the *Mi-1*-based resistance, SA application increases the effect of *R*-gene-based resistance in soybean to *A. glycines* whereas SA application to a susceptible soybean has no influence on aphid performance (Studham and MacIntosh, 2013). *Vat* gene function involves the JA, ET, and auxin signaling pathways but with a peroxidase burst soon after aphid attack. Upon aphid recognition by *Vat* in melon plants, peroxidase activity, and callose and lignin lining of the cell walls increase along the stylet path. When the aphids probe the plants they may be deterred by the oxidative burst in the plant cells or hindered to probe and feed efficiently by the cell wall thickening (Boissot et al., 2016). Certain short RNAs (microRNAs) with phytohormonal-related gene transcripts as targets have a role in this resistance reaction, for example by inactivating auxin receptors and signaling (Sattar and Thompson, 2016). The third aphid resistance gene sequenced so far, *SLI1*, codes for a sieve element-lining protein associated with organelles and proteins in the thin layer of cytoplasm along the phloem sieve tube plasma membrane, potentially making it more difficult for aphids to penetrate and reach the sieve element lumen, alternatively to cause aphid food canal occlusion (Kloth et al., 2017).

### Local and Systemic Induction Patterns Influencing Aphid Performance

It is common for aphids to induce plant resistance to subsequent aphid colonization at the systemic level (i.e. in leaves or tissue away from the infested site) whereas direction of local effects of previous infestation is generally the opposite, i.e. induced susceptibility, according to Zust and Agrawal (2016). This is in line with our expectations that aphids modify their feeding site to their advantage. Some of the studies in **Table 1** analyzed both types of tissues, in pairs of aphids and hosts representing compatible interactions. Not unexpectedly, gene regulation and metabolites in the infested tissue were more different from control than systemic tissue; as shown in *Arabidopsis* exposed to *M. persicae* (Kerchev et al., 2013), in black mustard infested by *B. brassicae* (Ponzio et al., 2017) as well as in wheat infested by *Diuraphis noxia* Kurdjumov (Liu et al., 2011), *S. avenae* (Guan et al., 2015), or *R. padi* (Greenslade et al., 2016). However, certain gene classes are sometimes found more activated in systemic than in infested tissue. In a wheat study with *S. avenae*, relatively more of proteins involved in metabolic processes were regulated in systemic than in infested tissue of a susceptible cultivar (Ferry et al., 2011). This may fit with the sink-source theory; that the aphids’ feeding site is a sink tissue that withdraws nutrients produced in non-infested plant tissue, as has been shown for nitrogen in alfalfa infested by *A. pisum* (Girousse et al., 2005).

Apart from nutritional aspects, defense-related aphid-induced plant moderations support the resistance/susceptibility pattern in systemic/infested leaves as suggested by Zust and Agrawal (2016). In wheat susceptible to *S. avenae*, a JA-regulated agglutinin was downregulated initially both in the infested and in the systemic tissue but after 8 days only in the infested tissue (Ferry et al., 2011). Several agglutinins, also called lectins, of plant origin have given negative effects on aphid performance when tested in aphid diets or transgenic plants (Vandenborre et al., 2011). Furthermore, *Myzus nicotianae* Blackman downregulated a germin gene in infested but not in systemic leaves of wild tobacco (Voelckel et al., 2004). Germin is an oxalate oxidase involved in release of H_2_0_2_- and Ca^2+^-mediated signaling, and in cross-linking of cell wall polymers (Lane et al., 1993). In systemic leaves of *Arabidopsis*, JA-induced anti-insect storage protein genes were upregulated as well as genes for cell-wall strengthening and synthesis of plant secondary compounds whereas in infested leaves, ABA and a redox-responsive transcription factor for ABA signaling were suggested to favor *M. persicae* reproduction (Kerchev et al., 2013) (**Table 1**).

However, there are studies of aphids on host plants where pre-infestation by conspecifics causes subsequent aphids to avoid or perform less well on the same leaves, possibly due to high infection pressure at pre-infestation (De Vos and Jander, 2009; Zust and Agrawal, 2016). The effects of induction by pre-infestation also vary depending on if plants are susceptible or resistant to the aphid. *R*-gene based resistance in barrel medic against the aphid *Acyrthosiphon kondoi* Shinji was induced by previous feeding by conspecifics, whereas no evidence of induced susceptibility was found in a susceptible host (Klingler et al., 2005). In peach susceptible to *M. persicae*, aphid performance was improved by pre-infestation whereas in various resistant peach genotypes, *M. persicae* pre-infestation either led to reduced, improved, or no change in aphid performance (Sauge et al., 2006). The pre-infestation effect is also dependant on aphid biotype; e.g. in barley with monogenic resistance to *S. graminum*, pre-infestation with a non-compatible aphid biotype led to reduced feeding by a compatible biotype, indicating defense induction by the former (Hays et al., 1999). Similarly, Zytynska et al. (2016) found biotype-dependant induction of numerous transcripts and reduction of aphid population growth of *S. avenae* in barley. In neither of these two barley studies were there aphid biotypes that induced susceptibility to another biotype though.

### Aphid Modification of Food Accessibility

To successfully access and feed on phloem sap, aphids need to penetrate the plant tissue with their mouthparts, prevent sieve tube clogging as a result of the mechanical damage they cause, and maintain leaf water potential in the vascular bundles.

In order for aphids to reach the phloem, their stylets first penetrate the epidermis in the cell wall between two cells and then proceed deeper between other cells in the plant tissues. Aphids also penetrate the cell walls when they probe cells along the pathway to the vascular bundles, as well as when they feed from phloem or drink from xylem (Pettersson et al., 2007). Thus there is reason to believe that the structure of the cell walls may influence the ease with which the phloem and xylem can be reached, especially by the small, newborn nymphs in a colony. The main component of the cell walls is cellulose which is combined with other polysaccharides; various hemicelluloses, pectin (galacturons) and callose (*β*-1,3-glucans), and glycoproteins (arabinogalactan proteins and extensins). The primary cell wall is flexible whereas the secondary cell wall that is deposited after cessation of cell expansion is rigid, strengthened by the polyphenol lignin (Zhong et al., 2019). Some of the studies listed in **Table 1** infer cell wall manipulations by aphids. Reddy et al. (2013) found some pectinase genes upregulated by *D. noxia* in susceptible compared to resistant wheat, indicating aphid modification of cell walls to its favor. Furthermore, an arabinogalactan protein gene was downregulated by *M. persicae* in *Arabidopsis* and the authors suggest that this may facilitate cell wall penetration by the aphid (Couldridge et al., 2007). Many expansin genes were found to be upregulated in another study of the same aphid/host combination (Bricchi et al., 2012). Expansins are proteins involved in cell wall loosening. In black mustard, the *O*-methyltransferase gene was downregulated by *B. brassicae* which might in turn lead to less lignin and loosened secondary cell walls (Broekgaarden et al., 2011) (**Table 1**).

Certain *β*-1,3-glucanases are among the pathogenesis-related (PR) proteins, induced *via* the SA defense pathways by various pathogens (van Loon and van Strien, 1999). *β*-1,3-Glucanase genes have been found upregulated by aphids as well. However, rather than contributing to plant defense, these enzymes may facilitate aphid feeding since they might reduce callose sealing of phloem tubes through breaking down callose. Pores in the phloem sieve plates are lined with callose collars that may regulate phloem sap flow from one cell to another. In undamaged phloem tissues there is a balance between build-up of callose by callose synthases and break-down by *β*-1,3-glucanases. When a sieve element is damaged, there is a rapid plugging of the sieve plate pores, within seconds to minutes. However, there are several examples of compatible aphid/host relationships where *β*-1,3-glucanases in the plants are upregulated by aphid infestation indicating that this is a way for these aphids to reduce or hinder phloem plugging (van Bel and Will, 2016), which otherwise might be permanent (Pirselova and Matusikova, 2013). Both *M. persicae* and *B. brassicae* upregulated *β*-1,3-glucanase gene expression in the susceptible host *Arabidopsis* (Moran et al., 2002). Zhu-Salzman et al. (2004) found the same in sorghum susceptible to *S. graminum*. Comparing two *R. padi*-susceptible and two partially resistant barley lines, Delp et al. (2009) found one glucanase gene upregulated in all four, one upregulated specifically in the two susceptible and one constitutively expressed only in the two susceptible lines with some upregulation by *R. padi*. Reddy et al. (2013) compared bulks of susceptible and resistant wheat lines and found that three *β*-1,3-glucanase genes were more upregulated by *S. graminum* in the susceptible than in the resistant bulk. Sun et al. (2018) found the same relationship between the *A. pisum* infestation and the glucanase regulation when comparing one partially resistant and one susceptible near-isogenic barrel medic line, so did Park et al. (2006) who compared sorghum genotypes resistant and susceptible to *S. graminum* (**Table 1**).

High leaf water potential facilitates xylem feeding which is a way for aphids to compensate for the high osmolarity of phloem sap (Sun et al., 2015). ABA signaling controls stomatal closure and leaf transpiration and aphid upregulation of this pathway might serve to maintain leaf water potential, as suggested in studies of corn infested by *Rhopalosiphum maidis* Fitch (Tzin et al., 2015) and of barrel medic by *A. pisum* (Sun et al., 2015). Aquaporins are proteins that facilitate water and other small molecules movement across cell membranes in either direction (Kapilan et al., 2018). In susceptible and moderately resistant cabbage, aquaporin genes were upregulated by *B. brassicae* (Broekgaarden et al., 2008) and in celery by *M. persicae* (Divol et al., 2005), something which in turn might increase water availability for the xylem and allocation of phloem sap.

Plant tissue infested by aphids functions as a sink to which photo-assimilates are directed (Hawkins et al., 1987). In many plant species the disaccharide sucrose is the predominant sugar transported in the phloem. Sucrose is produced in the green tissues during photosynthesis and its apoplastic cell-to-cell movement to the phloem is mediated by transporter and facilitator proteins. Transporters also help sugar uptake in sink tissue cells, in the form of sucrose or the monosaccharides glucose and fructose (Lemoine et al., 2013). A monosaccharide symporter gene was upregulated in *Arabidopsis* by *M. persicae* as well as by *B. brassicae*, suggesting that infested tissues function as a nutrient sink favorable to the aphids (Moran et al., 2002). The sink theory was also referred to by Delp et al. (2009) since two sugar transporter genes were upregulated locally by *R. padi* in both susceptible and partially resistant barley lines and another only in the susceptible lines. In cotton *A. gossypii* strongly induced a phosphate translocator gene that potentially increases sugar content in phloem sap as well as an asparaginase gene that increases the access to nitrogen (Dubey et al., 2013) (**Table 1**). Aphid control of plant genes related to sink effects deserves more attention in future studies.

### Aphid Modification of Food Quality

Phloem sap is imbalanced as aphid food since it is rich in sugars and relatively low in amino acids (Dinant et al., 2010). Genes for glutamate and glutamine synthases and a nitrate transporter were found to be upregulated by *M. nicotianae* in a wild tobacco host, more so in infested than in systemic leaves (Heidel and Baldwin, 2004; Voelckel et al., 2004). In tomato, *M. euphorbiae* upregulated nitrogen transporters genes (Coppola et al., 2013) and increased the N/C ratio (Rodriguez-Saona et al., 2010). A gene for nitrate reductase was upregulated in soybean susceptible to *A. glycines* (Prochaska et al., 2015) and in sorghum susceptible to *S. graminum* (Zhu-Salzman et al., 2004), but was downregulated in an aphid-resistant soybean (Prochaska et al., 2015). Nitrate reductase is required for nitrogen assimilation into amino acids. Furthermore, in addition to being low in amino acids in general, the amino acid composition of phloem sap is imbalanced for certain essential amino acids. Nutrient-focused studies have shown that *D. noxia* and *S. graminum* increased phloem concentrations of essential amino acids in susceptible barley and wheat (Telang et al., 1999; Sandström et al., 2000), even though the aphids’ obligate endosymbionts ensure that aphids are not dependant on the plant for provision of such amino acids (Baumann, 2005). Both of these aphid species cause typical leaf symptoms on susceptible hosts whereas *R. padi* infesting the same hosts does not, neither does it change the host amino acid profile. Among the non-essential amino acids, glutamine was significantly increased by *D. noxia* and *S. graminum*. This amino acid has been associated with phloem unloading sites but also senescing tissues (Sandström et al., 2000). In line with this, Dubey et al. (2013) found a senescence-related gene upregulated by *A. gossypii* in susceptible cotton and Tzin et al. (2015) showed cytokinin-related genes downregulated by *R. maidis* in susceptible corn resulting in lowered cytokinin concentration and thereby earlier senescence. However, comparing one susceptible and one moderately resistant pea genotype infested by *A. pisum*, Carrillo et al. (2014) found a relative decrease in a putative senescence-related protein in the susceptible genotype along with increase in proteins involved in photosynthesis, carbohydrate metabolism, and amino acid synthesis (**Table 1**).

Apart from nutrients, there is a large array of secondary compounds in the phloem sap, which might affect feeding aphids negatively depending on aphid host adaptation (Dinant et al., 2010; Foyer et al., 2015). *M. persicae* downregulated many genes related to flavonoid biosynthesis as well as transporter genes related to secondary metabolites in *Arabidopsis* (Bricchi et al., 2012) and potato (Alvarez et al., 2014), while *S. avenae* downregulated a lectin-like protein in wheat, previously inferred to have a role in resistance to Hessian fly (Ferry et al., 2011). Sanchez-Arcos et al. (2019) compared inductions by *A. pisum* biotypes adapted to alfalfa, clover, and pea and found that certain compounds, e.g. a triterpene saponin, were downregulated in alfalfa by the adapted biotype but not by the other two (**Table 1**).

Thus aphids have the capacity to modify the composition of phloem sap at their feeding site to their advantage.

### Components of Watery Saliva That May Facilitate Host Use

Several aphid effectors have now been shown to contribute to aphid virulence; C002 and Armet from *A. pisum* and *M. persicae*, Me10 and Me23 from *M. euphorbiae*, and Mp1, Mp2, and Mp55 from *M. persicae*; but their molecular interactions with the plants have just begun to be revealed (Rodriguez and Bos, 2013; Elzinga et al., 2014; Jaouannet et al., 2014; Rodriguez et al., 2017; Cui et al., 2019). Armet from *A. pisum* and *M. persicae* promotes SA accumulation (Cui et al., 2019). Mp1 from *M. persicae* hampers the Vacuolar Protein Sorting Associated Protein52 (VPS52) in the hosts *Arabidopsis* and potato. VPS52 vesicle trafficking is suggested to either contribute to plant defense or to reduce nutrient availability (Rodriguez et al., 2017). Mp55 when expressed in *Arabidopsis*, reduces callose deposition, hydrogen peroxide accumulation and glucosinolate conversion and increases host acceptance to *M. persicae* (Elzinga et al., 2014).

Other components of watery saliva may have a more direct influence on host plant suitability. Ca^2+^-binding proteins play a role in preventing sieve plate occlusion by protein and callose plugs and sieve element protein-lining. Proteases in aphid saliva may break down protein plugs in sieve plates and proteins in cytoplasm lining the sieve tube walls as well as degrade free proteins in the phloem sap, among them plant defense proteins. Proteases thereby also increase amino acid availability in phloem. Polyphenoloxidases, peroxidases, and oxidoreductases may reduce phenols and reactive oxygen species involved in plant defense (van Bel and Will, 2016; Kloth et al., 2017).

## Applications in Precision-Breeding for Resistance to Aphids

Plant genes that facilitate aphid host acceptance and performance can be called *S* genes, in line with the nomenclature for the corresponding genes in pathogen/plant interactions (van Schie and Takken, 2014). If an *S* gene is mutated so that it no longer functions favorably to the aphid the plant will be more resistant. Recessively inherited resistance genes may be such mutated susceptibility genes. Although most of the resistance genes characterized in aphid resistant germplasm are dominantly inherited there are a number of documented recessive genes; one in peanut associated with resistance to *Aphis craccivora* Koch, two in soybean against *A. glycines*, two in corn associated with resistance to *R. maidis*, and two introgressed in wheat; of which one causes resistance to *D. noxia* and the other to *S. graminum* (Dogimont et al., 2010). However, as far as we know none of these genes have been cloned and tested for their functions in aphid resistance. One well-known example of plant *S* genes in relation to pathogens is the recessive resistance to powdery mildew by *mlo* alleles which cause resistance to all races of that pathogen in barley. The exact function of the MLO protein is not known but it is involved in negative regulation of plant defense (Pavan et al., 2010; Kusch and Panstruga, 2017). Resistance breeding *via* mutagenesis or RNAi of *S* genes has been suggested for increasing resistance to plant pathogens and nematodes (De Almeida Engler et al., 2005; Pavan et al., 2010). Site-directed mutagenesis has been successful in introducing *mlo* resistance to powdery mildew in wheat, race-specific resistance to bacterial blight in rice and canker resistance in citrus for example. In the latter two examples, the promoter regions of the susceptibility genes were mutated. In yet another example, a gene for an ET responsive element that is a negative regulator of resistance to rice blast was mutated. Also resistance to several viral diseases has been achieved by plant-mediated gene mutations in the viral genome, or by mutations of *S* genes in the plant genome (Weeks et al., 2015; Mushtaq et al., 2019; Sedeek et al., 2019). In the following section, we will discuss the possibility to exploit knowledge about *S* genes for breeding resistance to aphids, namely to modify *S* gene function by site-directed mutagenesis or *via* RNAi.

Mutation techniques are now available that allow mutations specifically in target genes as opposed to the conventional chemical or radiation mutation methods, which generate mutations in non-targeted genes in addition to the targeted ones. The most recent and versatile technique by which to make such mutations is CRISPR/Cas9 (Clustered Regularly Interspaced Short Palindromic Repeat/CRISPR-associated protein9), a technique that mimics a microbe defense mechanism. There are different approaches for applying this technique, while the most common one is to knock out the target gene function. With this approach, a guide RNA sequence binds to a matching DNA sequence in the target gene and the Cas9 enzyme makes a double DNA strand break. The break is then repaired by the cell machinery and normally results in short insertion or deletion mutations which may result in knock-out of the gene function. Moreover, this technique is also possible to use for a specific single base pair editing, or overexpression or downregulation of target genes although these approaches are not yet as well developed and applied as the knock out approach (http://www.addgene.org/crispr/guide/; accessed on 3 July 2019). Alternatively, it is possible to use the RNAi technique to downregulate the gene expression. This method builds upon transformation of DNA constructs that give rise to short double-stranded RNA that after plant processing changes the plant transcriptome (Younis et al., 2014).

There are numerous examples of mutants produced with the older mutation techniques (i.e. by chemicals, radiation or T-DNA insertion) used in studies of gene function, often in model species such as *Arabidopsis* (De Vos et al., 2007; Louis and Shah, 2013). However, to be applied in aphid resistance breeding of crops it is necessary to test the effects in the target crop and with the target pest. Furthermore, it is essential to make sure that no additional mutations interfere. Existing mutants tested in earlier studies are, however, useful in providing indications of potential susceptibility mechanisms to exploit and also which general pleiotropic plant effects can be expected for a given gene knock-out (Thompson and Goggin, 2006; Huckelhoven et al., 2013; van Schie and Takken, 2014).

Since many studies have shown that the JA-related plant defense is functional for reducing host plant susceptibility to aphids and that aphids can counteract the induction of this pathway *via* upregulation of the SA-based defense, it is possible to exploit this knowledge for aphid resistance breeding by mutating genes in the SA defense signaling pathway so that the SA–JA cross-talk is disturbed and JA signaling can take place. However, this strategy may be risky because it will probably make the plant more susceptible to biotrophic pathogens, against which SA-induced defense is important. Besides, as described above, there is probably cross-talk also between other hormonal signaling pathways, which might be important for aphid-induced susceptibility, but at the same time important for other plant functions like tolerance to abiotic stresses.

Ideally the *S* gene candidate for mutation should be more specific to the aphid/plant interaction and have functions that are not absolutely vital for plant growth and development. This is possible if there are homologues of the gene differing in whether they are regulated by aphids or not. An example of this is *β*-1,3-glucanase genes, of which only some are upregulated by aphids, more so in the susceptible hosts (Park et al., 2006; Delp et al., 2009, Reddy et al., 2013; Mehrabi et al., 2016; Sun et al., 2018). Recently, Shoala et al. (2018) tested *Arabidopsis β*-1,3-glucanase mutants and proposed that one out of the four targeted *β*-1,3-glucanases is a susceptibility factor for *M. persicae*. We are currently testing barley with one or two of such genes mutated for potentially increased resistance to *R. padi*. The rationale for this attempt is that we expect less of glucanases and therefore more callose accumulating in the phloem in response to aphid infestation, which in turn may reduce aphid access to the phloem sap.

Another possibility to reduce susceptibility to aphids might be to hinder aphid-induced loosening of plant cell walls by knocking out or down genes associated with cell wall structure. Previous global gene expression studies have indicated that certain cell wall-related genes, which showed increased expression levels upon aphid infestation, such as pectinase (Reddy et al., 2013) and expansin genes (Bricchi et al., 2012), might be potential *S* genes to target by CRISPR/Cas9 or RNAi. One type of cell wall-related candidate *S* genes has already been confirmed *via* transient RNAi, i.e. (1,3;1,4)-*β*-glucanases that can degrade plant cell wall cellulose. Corresponding RNA sequences were shown to be induced to higher levels in susceptible than in resistant wheat lines 5 h after infestation by *D. noxia*. Knock-down of plant (1,3;1,4)-*β*-glucanase sequences increased plant resistance to *D. noxia,* suggesting that they are aphid-induced susceptibility factors (Anderson et al., 2014). Moreover, instead of directly targeting the *S* gene, a transcription factor gene with a regulatory role for the target gene might be knocked out or down. The transcription factor *WRKY22* that enhances the susceptibility to *M. persicae* in *Arabidopsis* can serve as an example. Aphids took longer time to penetrate the epidermis and mesophyll and reach the vascular bundles of the *wrky22* mutants, possibly due to the downregulation of the cell-wall loosening genes for pectin lyases and expansins (Kloth et al., 2016).

Genes involved in nutrient transport such as certain sugar and nitrogen transporters may be another type of genes to target. Like with some aphid examples mentioned above (**Table 1**), sugar transporters have been found to be regulated also by pathogens and disease resistance has been confirmed in the mutants with edited promoter regions or in genotypes with natural mutations in coding regions of sugar transporter genes (Julius et al., 2017;Sedeek et al., 2019). Based on the imbalanced N/C ratio of phloem sap, nitrogen-related genes are possibly more important to target for reducing aphid performance than carbohydrate-related genes. Genes for turgor-regulating proteins, aquaporins, offer another possibility for gene editing in order to indirectly reduce the aphid access to nutrients in the phloem.

## Concluding Remarks

Although several omics studies have been carried out in aphid-infested plants, the molecular mechanisms explaining aphid-induced plant susceptibility are still largely unknown. The fact that the global expression studies performed until now commonly include a single susceptible host genotype or compare solely one resistant and one susceptible genotype makes it difficult to draw conclusions about potential susceptibility roles of plant genes in relation to aphid performance. Therefore several representatives of resistant and susceptible plant genotypes should be compared in future studies in order to unravel more general patterns. Thus, there are so far only few candidate genes for induced plant susceptibility to aphid attack which might be exploited for improving crop resistance to aphids. We expect this field of research and plant resistance breeding to expand in the coming years.

Based on the knowledge we have so far, we have suggested certain types of potential susceptibility-associated genes for improving crop resistance to aphids. We also propose functional studies of recessively inherited resistance discovered in some aphid–crop combinations which might reveal currently unknown types of susceptibility genes that could be knocked out or down in other crops to improve their resistance to aphids. For application in commercial plant breeding, it will be necessary to find susceptibility genes that do not compromise the crop yield, or are at least so effective in controlling a severe pest that some crop loss can be accepted. Reluctance to accept crops developed by gene technology as for instance in EU is currently an obstacle for efficient reduction of host susceptibility as a resistance breeding approach.

## Author Contributions

IÅ wrote the main part of the manuscript. L-HZ suggested manuscript improvements and added suggestions for candidate gene types to target, along with S-YK.

## Conflict of Interest

The authors declare that the research was conducted in the absence of any commercial or financial relationships that could be construed as a potential conflict of interest.
